# Supplementary material for: The Influence of Age on Cerebral Tissue Oxygenation in Vasovagal Syncope and Orthostatic Hypotension
Source: J Clin Med. 2022 Jul 25;11(15):4302. doi: 10.3390/jcm11154302 (PMC9332367; doi:10.3390/jcm11154302)
Supplement: Supplementary file 1 [file jcm-11-04302-s001.zip › jcm-1697962-supplementary.pdf]

Supplementary Tables

Table S1. Medications among patients with vasovagal syncope, orthostatic hypotension and negative HUT patients.

	VVS	OH	Negative HUT
**Beta blockers**	10.1	19.8	17.1
**Loop diuretics**	0.7	7.4	3.7
**Hydrochlorothiazide**	2.9	3.3	2.4
**Spironolactone**	0.7	3.3	0*
**ACE**	2.9*	11.7*	14.6
**ARB**	7.2	21.5	4.9
**Alfa blockers**	0	2.5	1.2
**Calcium channel blockers**	5.8	17.4	6.1
**Antihypotensives**	0.7	6.6	2.4

Values are presented as percentage values. Abbreviations: VVS=vasovagal syncope, OH=orthostatic hypotension.

ACE=angiotensin converting enzyme, ARB=angiotensin receptor blocker, HUT=head-up tilt. *n=1 missing.

Table S2A. Association between cerebral tissue oxygen saturation, systolic blood pressure and age at examination in vasovagal syncope patients.

**A.**	Beta	p-value
SctO2 supine	-0.019	0.317
SctO2 3 min	-0.015	0.484
SctO2 10 min	-0.015	0.502
SctO2 presyncopal phase	-0.123	<0.001
SctO2 syncope	-0.034	0.200
SctO2 minimum	-0.010	0.628
Delta presyncope	0.104	<0.001
**B.**	Beta	p-value
SctO2 supine	-0.036	0.114
SctO2 3 min	-0.044	0.071
SctO2 10 min	-0.058	0.033
SctO2 presyncopal phase	-0.096	0.001
SctO2 syncope	-0.022	0.423
SctO2 minimum	-0.038	0.093
Delta presyncope	0.026	0.315
**C.**	**Beta**	**p-value**
SBP supine	0.494	<0.001
SBP 3 min	0.528	<0.001
SBP 10 min	0.601	<0.001
SBP presyncopal phase	-0.200	0.052
SBP minimum	0.488	<0.001
Delta presyncope	0.693	<0.001
**D.**	Beta	p-value
SBP supine	0.481	<0.001
SBP 3 min	0.536	<0.001
SBP 10 min	0.614	<0.001
SBP presyncopal phase	-0.049	0.628
Delta presyncope	0.507	<0.001

A: Univariable linear regression model including age (independent variable) and SctO2 (dependant variable.

B: Multivariable-adjusted linear regression model including age (independent variable) and SctO2 (dependant variable, adjusted for sex and concurrent systolic blood pressure (supine position or after 3 or10 min of head up tilt, mean of 45-15 seconds prior to syncope, SBP during syncope, SBP during lowest measured SctO2 prior to reflex activation or delta SBP from supine to presyncope).

C: Univariable linear regression model including age (independent variable) and SBP (dependant variable.

D: Multivariable-adjusted linear regression model including age (independent variable) and SBP (dependant variable, adjusted for sex and concurrent cerebral tissue oxygenation (supine position or after 3 or10 min of head up tilt, mean of 45-15 seconds prior to syncope, or delta SctO2 from supine to presyncope).

SBP=systolic blood pressure, SctO2=cerebral tissue oxygenation.

Table S2B. Association between cerebral tissue oxygen saturation, systolic blood pressure and age at examination in orthostatic hypotension patients.

**A.**	Beta	p-value
SctO2 supine	-0.043	0.024
SctO2 3 min	-0.058	0.004
SctO2 10 min	-0.081	<0.001
SctO2 presyncopal phase	-0.200	<0.001
SctO2 syncope	-0.071	0.051
SctO2 minimum	-0.089	<0.001
Delta presyncope	0.109	<0.001
**B.**	Beta	p-value
SctO2 supine	-0.067	0.005
SctO2 3 min	-0.078	0.001
SctO2 10 min	-0.097	<0.001
SctO2 presyncopal phase	-0.203	<0.001
SctO2 syncope	-0.079	0.057
SctO2 minimum	-0.112	<0.001
Delta presyncope	0.070	0.052
**C.**	Beta	p-value
SBP supine	0.650	<0.001
SBP 3 min	0.382	<0.001
SBP 10 min	0.374	0.001
SBP presyncopal phase	0.005	0.962
SBP minimum	0.164	0.091
Delta presyncope	0.702	<0.001
**D.**	Beta	p-value
SBP supine	0.741	<0.001
SBP 3 min	0.508	<0.001
SBP 10 min	0.556	<0.001
SBP presyncopal phase	-0.044	0.777
Delta presyncope	0.590	0.008

A: Univariable linear regression model including age (independent variable) and SctO2 (dependant variable.

B: Multivariable-adjusted linear regression model including age (independent variable) and SctO2 (dependant variable, adjusted for sex and concurrent systolic blood pressure (supine position or after 3 or10 min of head up tilt, mean of 45-15 seconds prior to syncope, SBP during syncope, SBP during lowest measured SctO2 prior to reflex activation or delta SBP from supine to presyncope).

C: Univariable linear regression model including age (independent variable) and SBP (dependant variable.

D: Multivariable-adjusted linear regression model including age (independent variable) and SBP (dependant variable, adjusted for sex and concurrent cerebral tissue oxygenation (supine position or after 3 or10 min of head up tilt, mean of 45-15 seconds prior to syncope, or delta SctO2 from supine to presyncope).

SBP=systolic blood pressure, SctO2=cerebral tissue oxygenation.

Table S2C. Association between cerebral tissue oxygen saturation, systolic blood pressure and age at examination in patients with negative head-up tilt.

**A.**	Beta	p-value
SctO2 supine	-0.055	0.069
SctO2 3 min	-0.019	0.567
SctO2 10 min	-0.028	0.367
SctO2 minimum	-0.032	0.329
Delta minimum	-0.018	0.448
**B.**	Beta	p-value
SctO2 supine	-0.085	0.010
SctO2 3 min	-0.057	0.121
SctO2 10 min	-0.056	0.100
SctO2 minimum	-0.046	0.164
Delta minimum	-0.041	0.068
**C.**	Beta	p-value
SBP supine	0.429	<0.001
SBP 3 min	0.462	<0.001
SBP 10 min	0.439	<0.001
SBP minimum	0.389	<0.001
Delta SBP minimum	0.041	0.499
**D.**	Beta	p-value
SBP supine	0.464	<0.001
SBP 3 min	0.472	<0.001
SBP 10 min	0.455	<0.001

A: Univariable linear regression model including age (independent variable) and SctO2 (dependant variable.

B: Multivariable-adjusted linear regression model including age (independent variable) and SctO2 (dependant variable, adjusted for sex and concurrent systolic blood pressure (supine position or after 3 or10 min of head up tilt, minimum SBP, or delta SBP from supine to minimum value). Delta minimum was defined as the difference in supine value and minimum SctO2 during head up tilt test.

C: Univariable linear regression model including age (independent variable) and SBP (dependant variable.

D: Multivariable-adjusted linear regression model including age (independent variable) and SBP (dependant variable, adjusted for sex and concurrent cerebral tissue oxygenation (supine position or after 3 or10 min of head up tilt or minimum SctO2. Delta minimum was defined as the difference in supine value and minimum SBP during head up tilt test.

SBP=systolic blood pressure, SctO2=cerebral tissue oxygenation.

Table S3A. Systolic blood pressure according to age in vasovagal syncope patients.

	**Below 30 years (n=36)**	**30-60 years (n=73)**	**Above 60 years (n=30)**	**p-value**
SBP supine	123.4±10.4	128.9±14.0	146.7±17.9	<0.001[α]
SBP 3min	124.9±10.8	130.9±15.4†	150.1±20.9	<0.001[α]
SBP 10 min	119.6±12.4§	128.7±13.9‡	147.2±19.0	<0.001[α]
SBP presyncopal phase	85.6±21.8	77.6±19.7	77.6±20.8	0.133
SBP minimum	111.7±17.8*	120.2±12.1*	134.8±17.8	<0.001
SBP delta presyncope	37.8±20.0	51.3±21.9	69.1±22.6	<0.001
SBP delta minimum	11.6±16.9*	8.6±8.7*	11.9±10.4	0.297

Continuous variables are expressed as mean ± standard deviation of the mean. P-values are from one way-ANOVA, unless otherwise indicated, for differences in mean SBP in patients below 30 years, between 30-60 years and over 60 years of age. Delta presyncope was defined as the difference in supine value to 45-15 seconds prior to syncope. Delta minimum was defined as the difference in supine value and minimum value prior to reflex activation. *n=1, †n=3, ‡n=5, §n=6 missing. SBP=systolic blood pressure, SctO2=cerebral tissue oxygenation.

α = p-values from Welch test.

Table S3B. Systolic blood pressure according to age in vasovagal syncope patients.

			P-value
**SBP supine**	<30	30-60	0.063 $^{\alpha}$
		>60	<0.001 $^{\alpha}$
	30-60	<30	0.063 $^{\alpha}$
		>60	<0.001 $^{\alpha}$
	>60	<30	<0.001 $^{\alpha}$
		30-60	<0.001 $^{\alpha}$
**SBP 3 min**	<30	30-60	0.057 $^{\alpha}$
		>60	<0.001 $^{\alpha}$
	30-60	<30	0.057 $^{\alpha}$
		>60	<0.001 $^{\alpha}$
	>60	<30	<0.001 $^{\alpha}$
		30-60	<0.001 $^{\alpha}$
**SBP 10 min**	<30	30-60	0.006 $^{\alpha}$
		>60	<0.001 $^{\alpha}$
	30-60	<30	0.006 $^{\alpha}$
		>60	<0.001 $^{\alpha}$
	>60	<30	<0.001 $^{\alpha}$
		30-60	<0.001 $^{\alpha}$
**SBP presyncopal phase**	<30	30-60	0.135
		>60	0.257
	30-60	<30	0.135
		>60	1.000
	>60	<30	0.257
		30-60	1.000
**SBP minimum**	<30	30-60	0.020

		>60	<0.001
	30-60	<30	0.020
		>60	<0.001
	>60	<30	<0.001
		30-60	<0.001
**SBP delta presyncope**	<30	30-60	0.007
		>60	<0.001
	30-60	<30	0.007
		>60	0.001
	>60	<30	<0.001
		30-60	0.001
**SBP delta minimum**	<30	30-60	0.429
		>60	0.995
	30-60	<30	0.429
		>60	0.404
	>60	<30	0.995
		30-60	0.404

P-values are from two way-ANOVA with Tukey's test, unless otherwise indicated, for differences in mean SBP in patients below 30 years, between 30-60 years and over 60 years of age. Delta presyncope was defined as the difference in supine value to 45-15 seconds prior to syncope. Delta minimum was defined as the difference in supine value and minimum value prior to reflex activation. SBP=systolic blood pressure, HUT=head-up tilt test. α = p-values from Games-Howell comparisons post hoc test.

Table S4A. Systolic blood pressure according to age in orthostatic hypotension patients.

	Below 30 years (n=13)	30-60 years (n=32)	Above 60 years (n=76)	p-value
SBP supine	116.1±13.9	132.9±13.7	150.3±22.0	<0.001$^{\alpha}$
SBP 3min	112.6±14.8*	122.0±10.4	131.9±25.5*	0.002$^{\alpha}$
SBP 10 min	103.5±16.6†	112.0±15.8†	121.6±24.4§	0.011$^{\alpha}$
SBP presyncopal phase	74.6±13.1‡	70.5±16.0||	73.0±13.1#	0.810
SBP minimum	91.5±11.6	99.6±11.8	99.1±24.0*	0.105$^{\alpha}$
SBP delta presyncope	41.0±14.2‡	61.8±14.7||	77.7±28.7#	0.001$^{\alpha}$
SBP delta minimum	24.6±8.2	33.3±13.4	51.5±22.0*	<0.001$^{\alpha}$

Continuous variables are expressed as mean ± standard deviation of the mean. P-values are from one way-ANOVA or Welch test, unless otherwise indicated, for differences in mean SBP in patients below 30 years, between 30-60 years and over 60 years of age. Delta presyncope was defined as the difference in supine value to 45-15 seconds prior to syncope. Delta minimum was defined as the difference in supine value and minimum value prior to reflex activation. *n=1, †n=3, ‡n=6, §n=8, ||n=20, #n=52 missing. SBP=systolic blood pressure. α = p-values from Welch test.

Table S4B. Systolic blood pressure according to age in orthostatic hypotension patients.

			**P-value**
**SBP supine**	<30	30-60	0.004 $^{\alpha}$
		>60	<0.001 $^{\alpha}$
	30-60	<30	0.004 $^{\alpha}$
		>60	<0.001 $^{\alpha}$
	>60	<30	<0.001 $^{\alpha}$
		30-60	<0.001 $^{\alpha}$
**SBP 3 min**	<30	30-60	0.138 $^{\alpha}$
		>60	0.003 $^{\alpha}$
	30-60	<30	0.138 $^{\alpha}$
		>60	0.014 $^{\alpha}$
	>60	<30	0.003 $^{\alpha}$
		30-60	0.014 $^{\alpha}$
**SBP 10 min**	<30	30-60	0.357 $^{\alpha}$
		>60	0.022 $^{\alpha}$
	30-60	<30	0.357 $^{\alpha}$
		>60	0.060 $^{\alpha}$
	>60	<30	0.022 $^{\alpha}$
		30-60	0.060 $^{\alpha}$
**SBP presyncopal phase**	<30	30-60	0.815
		>60	0.961
	30-60	<30	0.815
		>60	0.873
	>60	<30	0.961
		30-60	0.873
**SBP minimum**	<30	30-60	0.107 $^{\alpha}$

		>60	0.185[α]
	30-60	<30	0.107[α]
		>60	0.988[α]
	>60	<30	0.185[α]
		30-60	0.988[α]
**SBP delta presyncope**	<30	30-60	0.024[α]
		>60	<0.001[α]
	30-60	<30	0.024[α]
		>60	0.087[α]
	>60	<30	<0.001[α]
		30-60	0.087[α]
**SBP delta minimum**	<30	30-60	0.033[α]
		>60	<0.001[α]
	30-60	<30	0.033[α]
		>60	<0.001[α]
	>60	<30	<0.001[α]
		30-60	<0.001[α]

P-values are from two way-ANOVA with Tukey's post hoc test, unless otherwise indicated, for differences in mean SBP in patients below 30 years, between 30-60 years and over 60 years of age. Delta presyncope was defined as the difference in supine value to 45-15 seconds prior to syncope. Delta minimum was defined as the difference in supine value and minimum value prior to reflex activation. SBP=systolic blood pressure, HUT=head-up tilt test. α = p-values from Games-Howell comparisons post hoc test.

Table S5A. Cerebral tissue oxygenation during HUT according to age in vasovagal syncope patients.

			**P-value**
**SctO2 supine**	<30	30-60	0.242
		>60	0.833
	30-60	<30	0.242
		>60	0.666
	>60	<30	0.833
		30-60	0.666
**SctO2 3 min**	<30	30-60	0.950
		>60	0.992
	30-60	<30	0.950
		>60	0.988
	>60	<30	0.992
		30-60	0.988
**SctO2 10 min**	<30	30-60	0.967
		>60	0.988
	30-60	<30	0.967
		>60	0.905
	>60	<30	0.988
		30-60	0.905
**SctO2 presyncopal phase**	<30	30-60	0.147
		>60	0.006
	30-60	<30	0.147
		>60	0.175
	>60	<30	0.006
		30-60	0.175

**SctO2 syncope**	<30	30-60	0.853
		>60	0.949
	30-60	<30	0.853
		>60	0.987
	>60	<30	0.949
		30-60	0.987
**SctO2 minimum**	<30	30-60	0.998
		>60	0.976
	30-60	<30	0.998
		>60	0.981
	>60	<30	0.976
		30-60	0.981
**SctO2 delta presyncope**	<30	30-60	0.563
		>60	0.003
	30-60	<30	0.563
		>60	0.015
	>60	<30	0.003
		30-60	0.015
**SctO2 delta syncope**	<30	30-60	0.824 [α]
		>60	0.993 [α]
	30-60	<30	0.824 [α]
		>60	0.808 [α]
	>60	<30	0.993 [α]
		30-60	0.808 [α]
**SctO2 delta minimum**	<30	30-60	0.090 [α]
		>60	0.566 [α]
	30-60	<30	0.090 [α]
		>60	0.586 [α]

	>60	<30	0.566 [α]
		30-60	0.586 [α]

P-values are from two way-ANOVA with Tukey's test, unless otherwise indicated, differences in mean SctO2 in patients below 30 years, between 30-60 years and over 60 years of age. Delta presyncope was defined as the difference in supine value to 45-15 seconds prior to syncope. Delta syncope was defined as the difference in supine value and lowest measured SctO2 during syncope. Delta minimum was defined as the difference in supine value and minimum value prior to reflex activation. HUT=head-up tilt test, SctO2=cerebral tissue oxygenation.

α = p-values from Games-Howell comparisons post hoc test.

Table S5B. Cerebral tissue oxygenation during HUT according to age in orthostatic hypotension patients.

			P-value
**SctO2 supine**	<30	30-60	0.575
		>60	0.111
	30-60	<30	0.575
		>60	0.392
	>60	<30	0.111
		30-60	0.392
**SctO2 3 min**	<30	30-60	0.614
		>60	0.085
	30-60	<30	0.614
		>60	0.230
	>60	<30	0.085
		30-60	0.230
**SctO2 10 min**	<30	30-60	0.635
		>60	0.010
	30-60	<30	0.635
		>60	0.009
	>60	<30	0.010
		30-60	0.009
**SctO2 presyncopal phase**	<30	30-60	0.032
		>60	<0.001
	30-60	<30	0.032
		>60	0.049
	>60	<30	<0.001
		30-60	0.049

**SctO2 syncope**	<30	30-60	0.319
		>60	0.187
	30-60	<30	0.319
		>60	0.977
	>60	<30	0.187
		30-60	0.977
**SctO2 minimum**	<30	30-60	0.708
		>60	0.006
	30-60	<30	0.708
		>60	0.004
	>60	<30	0.006
		30-60	0.004
**SctO2 delta presyncope**	<30	30-60	0.188
		>60	0.003
	30-60	<30	0.188
		>60	0.151
	>60	<30	0.003
		30-60	0.151
**SctO2 delta syncope**	<30	30-60	0.960
		>60	0.923
	30-60	<30	0.960
		>60	0.686
	>60	<30	0.923
		30-60	0.686
**SctO2 delta minimum**	<30	30-60	0.999
		>60	0.181
	30-60	<30	0.999
		>60	0.029

	>60	<30	0.181
		30-60	0.029

P-values are from two way-ANOVA with Tukey's multiple comparisons post hoc test for differences in mean SctO2 in patients below 30 years, between 30-60 years and over 60 years of age. Delta presyncope was defined as the difference in supine value to 45-15 seconds prior to syncope. Delta syncope was defined as the difference in supine value and lowest measured SctO2 during syncope. Delta minimum was defined as the difference in supine value and minimum value prior to reflex activation. HUT=head-up tilt test, SctO2=cerebral tissue oxygenation.

Table S5C. Cerebral tissue oxygenation according to age in negative head-up tilt tests.

			P-value
**SctO2 supine**	<30	30-60	0.588
		>60	0.207
	30-60	<30	0.588
		>60	0.572
	>60	<30	0.207
		30-60	0.572
**SctO2 3 min**	<30	30-60	0.980
		>60	0.848
	30-60	<30	0.980
		>60	0.901
	>60	<30	0.848
		30-60	0.901
**SctO2 10 min**	<30	30-60	0.998
		>60	0.665
	30-60	<30	0.998
		>60	0.638
	>60	<30	0.665
		30-60	0.638
**SctO2 minimum**	<30	30-60	0.980
		>60	0.805
	30-60	<30	0.980
		>60	0.649
	>60	<30	0.805
		30-60	0.649
**SctO2 delta minimum**	<30	30-60	0.302

		>60	0.540
	30-60	<30	0.302
		>60	0.980
	>60	<30	0.540
		30-60	0.980

P-values are from two way-ANOVA with Tukey's test for differences in mean SctO2 in patients below 30 years, between 30-60 years and over 60 years of age. Delta minimum was defined as the difference in supine value and minimum value prior to reflex activation. SctO2=cerebral tissue oxygenation.

Table S6. Cerebral tissue oxygenation according to age in negative head-up tilt tests.

	Below 30 years (n=23)	30-60 years (n=41)	Above 60 years (n=18)	p-value
SctO2 supine	72.8±4.8†	71.5±4.5	71.1±5.5	0.236
SctO2 3min	69.7±5.5	69.5±5.4	68.8±6.0	0.855
SctO2 10 min	70.0±5.2	69.9±5.0	68.6±5.5	0.619
SctO2 minimum	66.0±5.5†	66.2±4.8	64.9±5.7*	0.672
SctO2 delta minimum	6.8±3.2‡	5.3±3.6	5.5±4.3*	0.322

Continuous variables are expressed as mean ± standard deviation of the mean. P-values are from one way-ANOVA for differences in mean SctO2 in patients below 30 years, between 30-60 years and over 60 years of age. Delta minimum was defined as the difference in supine value and minimum value prior to reflex activation. *n=1, †n=2, ‡n=3 missing. SctO2=cerebral tissue oxygenation.

Table S7. Proportion of smokers according to age.

	<30 years	30-60 years	>60 years	p-value
VVS smokers (%)	6(16.7)	8(11.0)	1(3.3)	0.756
OH smokers (%)	2(15.4)	6(18.8)	10(13.2)	0.220
Negative HUT smokers (%)	9(39.1)	12(29.3)	1(5.9)	0.059*
Total smokers (%)	17(23.6)	26(17.8)	12(9.8)	0.031*

Data is defined as number of patients (percentage). P-values are from Chi-2 test. *N=1 missing. Abbreviations: VVS, vasovagal syncope. OH, orthostatic hypotension. HUT, head-up tilt test.

Table S8. Association between smoking and supine cerebral tissue oxygenation among VVS, OH and negative HUT patients.

	Smoker	Non-smoker	p-value
Scto2 supine	70.5	71.4	0.156*

Data is defined as mean supine SctO2 for VVS, OH and negative HUT patients (total=342 patients). P-values are from independent samples t-test. *N=3 missing. Abbreviations: SctO2, cerebral tissue oxygenation. VVS, vasovagal syncope. OH, orthostatic hypotension. HUT, head-up tilt test.
